# Body composition study by dual-energy x-ray absorptiometry in familial partial lipodystrophy: finding new tools for an objective evaluation

**DOI:** 10.1186/1758-5996-4-40

**Published:** 2012-08-31

**Authors:** Cynthia M Valerio, Lenita Zajdenverg, José Egídio P de Oliveira, Patricia B Mory, Regina S Moyses, Amélio F Godoy-Matos

**Affiliations:** 1 Metabolism Unit, Instituto Estadual de Diabetes e Endocrinologia, Rio de Janeiro and Catholic University, Rio de Janeiro, Brazil; 2 Department of Nutrology, Universidade Federal do Rio de Janeiro, Rio de Janeiro, RJ, Brazil; 3 Division of Endocrinology, Universidade Federal de Sao Paulo, São Paulo, SP, Brazil; 4 Instituto Estadual de Diabetes e Endocrinologia, Rua Moncorvo Filho 90 – Centro, Rio de Janeiro, RJ CEP 20211-340, Brasil

**Keywords:** Body composition, Dual-Energy X-ray Absorptiometry (DXA), Lipodystrophy

## Abstract

**Background:**

Familial partial lipodystrophies (FPLD) are clinically heterogeneous disorders characterized by selective loss of adipose tissue, insulin resistance and metabolic complications. Until genetic studies become available for clinical practice, clinical suspicion and pattern of fat loss are the only parameters leading clinicians to consider the diagnosis. The objective of this study was to compare body composition by dual energy X-ray absorptiometry (DXA) in patients with FPLD and control subjects, aiming to find objective variables for evaluation of FPLD.

**Methods:**

Eighteen female patients with partial lipodystrophy phenotype and 16 healthy controls, matched for body mass index, sex and age were studied. All participants had body fat distribution evaluated by DXA measures. Fasting blood samples were obtained for evaluation of plasma leptin, lipid profile and inflammatory markers. Genetic studies were carried out on the 18 patients selected that were included for statistical analysis. Thirteen women confirmed diagnosis of Dunnigan-type FPLD (FPLD2).

**Results:**

DXA revealed a marked decrease in truncal fat and 3 folds decrease in limbs fat percentage in FPLD2 patients (p <0.001). Comparative analysis showed that ratio between trunk and lower limbs fat mass, characterized as Fat Mass Ratio (FMR), had a greater value in FLPD2 group (1.86 ± 0.43 vs controls 0.93 ± 0.10; p <0.001) and a improved accuracy for evaluating FPLD2 with a cut-off point of 1.2. Furthermore, affected women showed hypoleptinemia (FLPD2 4.9 ± 2.0 vs controls 18.2 ± 6.8; p <0.001), insulin resistance and a more aggressive lipid profile.

**Conclusion:**

In this study, assessment of body fat distribution by DXA permitted an objective characterization of FLPD2. A consistent pattern with marked fat reduction of lower body was observed in affected patients. To our knowledge this is the first time that cut-off values of objective variables were proposed for evaluation of FPLD2.

## Background

Familial partial lipodystrophies (FPLD) are a clinically heterogeneous group of genetic disorders characterized by variable loss of subcutaneous adipose tissue in extremities. Affected patients exhibit insulin resistance and metabolic complications [1,2]. Usually diagnosis is delayed and cardiovascular disease is already established. Inappropriate fat distribution, not only reduction in the total amount of adipose tissue, also contributes to the metabolic state [3]. Recently, genetic research has improved our understanding on the mechanisms underlying lipodystrophies, but some patients with inherited forms do not have mutations in the known lipodystrophy genes [4].

Until genetic studies become available for clinical practice, metabolic features and the pattern of adipose tissue loss are the only parameters that may lead clinicians to consider the diagnosis. To date few studies have compared regional fat distribution in FPLD, most using Computed Tomography or Magnetic Resonance Images [5-7], which are not applicable for clinical practice. We have previously suggested that DXA might be a tool for this purpose [8,9].

The ratio between truncal and lower limbs fat evaluated by DXA, Fat Mass Ratio (FMR), has been proposed as a tool for recognizing HIV-related lipodystrophy [10-12]. We previously reported the use of FMR in distinct forms of lipodystrophy phenotypes, non-related to HIV infection [8,9]. Now, we have increased the number of studied patients and limited it to Familial Partial Lipodystrophy (FPLD).

## Patients and methods

In this study, we initially identified 18 women with partial lipodystrophy phenotype selected from Metabolism Unit of the State Institute of Diabetes and Endocrinology (Rio de Janeiro, Brazil) using criteria from previous reports of the syndrome [1,2]. All of them presented at least 3 of the following features: postpuberal loss of adipose tissue affecting lower limbs and sparing face and neck (essential criteria) with prominent veins and muscularity, acanthosis nigricans, polycystic ovarian syndrome (POS), hypertriglyceridemia and/or low high-density-lipoprotein (HDL)-cholesterol, diabetes mellitus type 2 (DM2) or impaired fasting glucose (IFG). POS was defined by presence of oligomenorrhea and hirsutism with no other known cause. DM2 was identified as two fasting glucose ≥ 126 mg/dl or use of antidiabetic agents and IFG was characterized as fasting glucose ≥ 100 mg/dl or ≥ 140 mg/dl after oral glucose tolerance test. Hypertriglyceridemia was diagnosed with triglycerides ≥150 mg/dl and low HDL-cholesterol level was <50 mg/dl.

Exclusion criteria adopted were: age under 12 years, pregnancy or breastfeeding, presence of acquired lipodistrophy (auto-immune or related to HIV infection or use of Highly Active Antiretroviral Therapy), severe renal or hepatic diseases, depression and alcoholism.

A control group with 16 healthy volunteers was matched for age, sex and BMI with lipodystrophic group. This group was recruited from outpatient clinic and hospital employees and was not related to the patients.

Diagnosis of Dunnigan-type FPLD (FPLD2) was confirmed by molecular analysis of LMNA gene provided by the Molecular Endocrinology Laboratory of the Universidade Federal de Sao Paulo. Thirteen women confirmed diagnosis of FPLD2.

The Ethics Committee of the Institution approved the protocol. A written informed consent was obtained from each patient, after the procedures involved in the study were fully explained.

### Genetic studies

Screening for mutations of LMNA was done through direct sequencing. Genomic DNA was extracted from peripheral blood, with the commercial kit (Gentra Puregene Blood Kit, Qiagen, Santa Clarita, CA, USA). Exons 1–12 and the intron-exon boundaries of LMNA gene were amplified by PCR using 14 pairs of primers. The PCR products were directly sequenced with the use of BigDye® Terminator Cycle Sequencing Kit version 3.1 and analysed by ABI PRISM 3100 Genetic Analyzer (Applied Biosystems, CA, USA).

### Anthropometrical examination

All participants were carefully examined by the same endocrinologist and had the following anthropometrical data registered: Body Weight (Kg), Height (m), Body Mass Index (BMI), waist circumference (WC), waist-to-hip ratio (WHR) and blood pressure. BMI was calculated as weight in kilograms divided by the square of height in meters (kg/m^2^). Waist circumference was determined at the midpoint between the lowest rib and the iliac crest in cm. WHR was defined as the ratio of waist girth to the largest circumference of the hips, measured at the trochanter major.

### Laboratory evaluation

Blood was collected after a 12 hour overnight fast in appropriate tubes and assays for plasma leptin, glucose, and lipoproteins. Plasma glucose was determined by glucose-oxidase method. Cholesterol, lipoproteins fractions and triglycerides were measured enzymatically. Plasma leptin was measured by Radioimmunoassay (Linco Research, Missouri, USA).

### Body fat analysis

DXA measurements of whole body, truncal and upper limbs fat mass, as well as lower limbs fat mass were obtained by DXA scan (LUNAR PRODIGY ADVANCE software version 9.5, LNR 41569 model, GE Medical Systems, Waukesha, WI). Central to peripheral fat Ratio (or Fat Mass Ratio [FMR]) was used to investigate body fat distribution [8,9]. Whole body DXA scans were obtained using manufacturer’s recommendation for subject positioning, scan protocols and scan analysis.

### Statistical analysis

Statistical analysis was performed with SAS software 6.11 (SAS Institute, Inc., Cary, North Carolina, USA). Unpaired t test was used for parametric variables and Mann–Whitney for non-parametric variables. Statistical analysis of subgroup with confirmed LMNA mutations (FPLD2) and comparisons with the control group were performed. The level of statistical significance was 5%. The sensitivity and specificity of FMR in patients with FPLD2 were calculated using receiver operating characteristic curve (ROC) analysis.

## Results

Genetic studies were carried out in the 18 female patients with partial lipodystrophy phenotype. The thirteen women that confirmed diagnosis of Dunnigan-type FPL (FPLD2) belonged to six different families. Family C and E contributed with one case each; Family B with a mother and her daughter; Family F and A with 2 and 3 sisters, respectively; and family D with 2 sisters and 2 cousins. All patients had a missense mutation in LMNA gene: nine patients harbored the heterozygous variation p. R482W; in three patients the mutation identified was p.R482Q (c.1445 G > A) and one patient exhibited a novel heterozygous variant in exon 8 (p.N466D) that we previously reported [13]. The 5 remaining women that underwent molecular biology studies showed no mutations in the LMNA gene. They have not yet been genetically tested for other lipodystrophy familial syndromes.

All of the FPLD2-affected women had acanthosis nigricans and eight had the diagnosis of DM2. One is on insulin therapy. Clinical features and metabolic status are reported in Table 1. Nine out thirteen of LMNA-mutated women had clinical phenotype of POS and seven exhibited hypertension.

**Table 1:** Characteristics of study group subjects

**Patient**	**Age (y)**	**LMNA mutation**	**BMI (kg/m2)**	**Clinical lipoatrophy**	**Fat deposition**	**DM2 (age of onset)**	**Dyslipidemia**	**POS**	**Comorbidities**	**FMR**
*A-I	44.5	p.R482Q	22,6	lower limbs	face, neck	IFG	low HDL-c, Hypertrigl.	yes	HBP	1,8
*A-II	42.9	p.R482Q	22,6	four limbs	face, neck	38	low HDL-c	no	HBP, CAD	2,38
*A-III	46.6	p.R482Q	23,1	lower limbs	face, neck	no	low HDL-c, Hypertrigl.	yes	HBP	1,91
**B-I	14.7	p.R482W	22,1	slight, lower limbs	no	no	no	yes	no	1,33
**B-II	27.9	p.R482W	27,6	four limbs	face, neck, supraclavicular	25	low HDL-c, Hypertrigl.	yes	no	1,71
C-I	26.3	p.R482W	27,4	four limbs	face, neck, supraclavicular	26	low HDL-c, Hypertrigl.	yes	Dilated Miocardiopathy	2,53
***D-I	28.4	p.R482W	24,7	four limbs	face, neck	22	low HDL-c, Hypertrigl.	yes	HBP	2,50
***D-II	31	p.R482W	20,5	slight, lower limbs	no	29	low HDL-c, Hypertrigl.	no	HBP	1,81
***D-III	33	p.R482W	25,1	slight, lower limbs	face, neck	no	low HDL-c, Hypertrigl.	yes	no	1,87
***D-IV	25.5	p.R482W	18,7	slight, lower limbs	no	no	Hypertrigl.	no	no	1,28
E	19.4	p.N466D	28,1	four limbs, abdome	face, neck	18	low HDL-c, Hypertrigl.	yes	HBP	1,62
****F-I	42	p.R482W	24,2	lower limbs	trunk, face, neck	31	low HDL-c, Hypertrigl.	yes	HBP	2,25
****F-II	51.3	p.R482W	18,5	lower limbs	no	28	low HDL-c, Hypertrigl.	no	muscular dystrophy	1,3
G	38.7	no	45,5	lower limbs	trunk	24	DM2, HDL-c,Hypertrigl	Yes	HBP	1,03
H	57.6	no	32,3	lower limbs	trunk	27	DM2, HDL-c,Hypertrigl	No	HBP	1,35
I	60.2	no	34,3	lower limbs	trunk	no	HDL-c,Hypertrigl	yes	HBP	1,04
J	56.8	no	32,5	lower limbs	trunk	43	HDL-c,Hypertrigl	yes	HBP	1,64
K	48.6	no	31,1	Four limbs	Trunk, neck	23	HDL-c,Hypertrigl	Yes	HBP	1,69

y- years.

FPLD- Familial Partial Lipodystrophy.

Low HDL-c- low HDL-cholesterol.

Hypertrig- Hypertriglyceridemia.

POS- Polycystic Ovarian Syndrome.

DM2- Type 2 Diabetes Mellitus.

IFG- Impaired Fasting Glucose.

CAD- Coronary Arterial Disease.

HBP- High Blood Pressure.

FMR- Fat Mass Ratio.

* Patients A-I, A-II and A-III are sisters and presented familial antecedents of a DM2, CAD and consanguinity.

** Patients B-I and B-II are mother and child.

***Patients D-I, D-II are siblings and cousins from D-III and D-IV. They present another relatives with partial lipodistrophy.

**** Patients F-I and e F-II are sisters and do not present another relatives with partial lipodistrophy.

**Figure 1:**
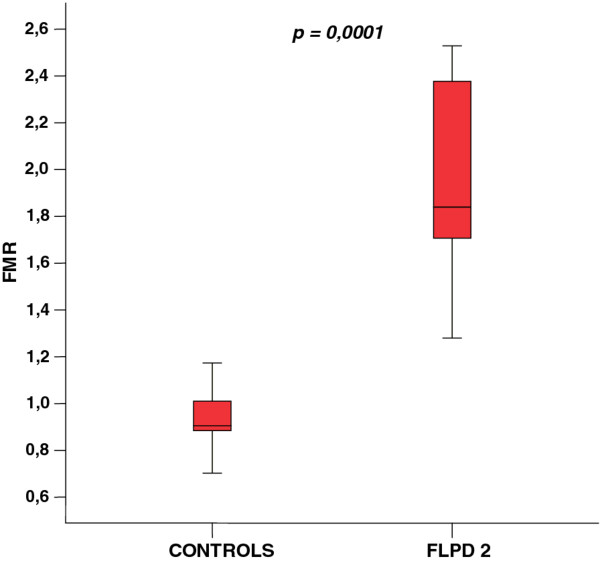
Comparison of distribution of Fat Mass Ratio (+/- SD) in control group and FLPD2 group.

Table 2 presents the comparison of control group and FPLD2 subjects. Despite the same age and BMI, lipodystrophic group had significant differences in fat distribution as evaluated by DXA. A marked decrease in total, as well as lower limbs fat mass was observed in affected patients. On reverse, a substantial trunk fat deposition was observed. Comparative analysis showed a greater value of FMR in FLPD2 group (1.86 ± 0.43 vs controls 0.93 ± 0.10; p <0.001) and improved accuracy for evaluating FPLD2 with a cut-off point of 1.2. According to values obtained by the ROC curve, the sensitivity and specificity using these cut-off values were 88.9 and 93.8%, respectively. Comparison of FMR in both groups is shown in Figure 1.

**Table 2:** Comparison of Anthropometric and DXA Measurements and Laborattory Evaluation in Patients with Familial Partial Lipodystrophy (FPLD), Familial Partial Lipodystrophy Dunnigan-type (FPLD2) and Control Group

	**Control (n = 16)**	**FLPD (n = 18)**	***p***	**FLPD2 (n = 13)**	***p***
Age (years)	40.6 ± 12.4	39.2 ± 14.5	0.73	33.3 ± 11.2	0.14
Waist (cm)	81.9 ± 9.2	82.3 ± 5.2	0.91	81.0 ± 7.2	0.89
Hip (cm)	103.1 ± 7.2	88.3 ± 5.4	<0.001	89.5 ± 5.0	<0.001
BMI (kg/m2)	25.6 ± 3.87	26.7 ± 6.6	0.76	23.5 ± 3.1	0.19
WHR	0.79 ± 0.06	0.94 ± 0.09	<0.001	0.90 ± 0.07	<0.001
Android/gynoid Fat Ratio	0.925 ± 0.1	1.177	<0.001	1.154	0.003
Total Fat (%)	43.6 ± 5.6	25.1 ± 11.0	<0.001	19.4 ± 4.9	<0.001
Troncular Fat (%)	44.2 ± 6.9	29.7 ± 11.0	<0.001	24.5 ± 6.2	<0.001
Upper Limbs Fat (%)	40.4 ± 5.2	22.9 ± 14.4	0.004	14.6 ± 5.1	<0.001
Lower Limbs Fat (%)	47.3 ± 5.6	19.4 ± 11.6	<0.001	13.7 ± 4.4	<0.001
Fat Mass (g)	28.5 ± 6.2	18.0 ± 12.0	0.002	11.7 ± 3.0	<0.001
Fat Mass Ratio (FMR)	0.93 ± 0.10	1.72 ± 0.46	<0.001	1.86 ± 0.43	<0.001
Leptin	18.1 ± 6.7	8.4 ± 9.0	<0.001	4.4 ± 2.1	<0.001
HDL Cholesterol	61.5 ± 13.4	39.4 ± 10.6	<0.001	36.5 ± 10.6	<0.001
LDL Cholesterol	126.3 ± 35.7	106.8 ± 38.9	0.20	110.7 ± 35.1	0.27
Triglycerides	109.3 ± 40.5	226.2 ± 115.3	<0.001	237.8 ± 130.6	<0.001

BMI = Body Mass Index; WHR = Waist-to-Hip Ratio.

Furthermore, FPLD2-affected women showed hypoleptinemia (median of 4.4 vs 18.1 ng/ml in controls), insulin resistance (8 out 13 had diabetes type 2 and two had impaired fasting glucose) and an aggressive lipid profile with lower levels of HDL-cholesterol (median of 36,5 versus 61,5 mg/dl in controls) and hypertriglyceridemia (p = 0.001).

## Discussion

In this study, final diagnosis of FPLD2 was based on genetic study. Once objective physical and metabolic features that reliably define FPLD have not been identified, we have used clinical and metabolic criteria described from previous reports of the syndrome to initially select our patients [1,2].

The main objective of this study was to find out if body composition parameters utilizing DXA would be useful for an objective evaluation of FPLD-affected subjects. Indeed, our data revealed a consistent pattern of adipose tissue distribution, with marked fat reduction of lower extremities and gluteal region (Table 2). This is in agreement with what has been described in several series of HIV patients [10-12]. Although scarcity of truncal fat may not be a feature of FPLD2, it is noteworthy that even when compared with BMI and age-matched controls, affected group presented a marked reduction of truncal body fat (44, 2 vs. 24, 5%). Interestingly, even not harboring increased truncal fat, these patients are severely metabolically compromised, suggesting that the lack of adipose tissue in the lower body is the main feature for metabolic abnormalities. Indeed, studies have suggested such a role for the peripheral fat in non-lipodystrophic subjects [14-16]. Van Pelt et al. [14] concluded that leg fat mass is associated with reduced CVD risk, independently of increased risk attributable to trunk fat mass. Tankó et al. [15] reached the same conclusion in a study using DXA for measuring fat distribution. In further support, Rocha et al. [16] revealed independent and opposite association of hip circumference with insulin resistance and atherothrombotic markers. Taken together, these findings underscore the metabolic protective effect of lower-body adiposity.

We previously reported the use of FMR for identification of all lipodystrophy phenotypes in a smaller series of patients [8]. Now, we have increased the number of studied patients and limited it to FLPD. Looking at the Table 1, one can realize that only 3 patients with FPLD2 had FMR under 1.6. They were leaner and exhibited an atypical form of FPLD2. Patient F-II, for example, is a small lady, measuring 1.45 m, BMI 18,5 kg/m2 suffering of a concomitant muscular dystrophy. On the other hand, all the patients without LMNA mutation presented FMR under 1.7 and 2 out 5 presented FMR within normal range. This finding suggests that FPLD2 subjects present higher values of FMR. Further studies with larger samples are warranted to better characterize body fat distribution in different types of FPLD.

In opposition to another anthropometric variables like waist-to-hip circumference ratio or android-gynoid percentual fat ratio, FMR was the only densitometry parameter with a well-defined cut off-point (>1,2) and great diagnostic accuracy for FLPD2. To our knowledge this is the first series where FMR cut-off values were proposed for evaluation of FPLD2. Bonnet et al. [10] were the first authors to suggest the use of FMR in HIV-related lipodystrophic patients, with a cut-off point of 1.3 +/−0.2. Recently, another series performed in HIV-infected men presented FMR >1.5 not only as a diagnostic criteria, but also for assessment of clinical evolution of lipodystrophy [11]. Therefore, our findings are in concordance with data derived from HIV-related lipodystrophy and point to the need of more studies in FLPD.

Patients with FPLD also need to be distinguished from Köbberling variety of FPLD (FLPD1). On clinical basis, the loss of adipose tissue in the Köberling variety is said to be only restricted to extremities [17,18]. Patients have normal amounts of fat in face and may have normal, or even excess, subcutaneous truncal fat, as observed in the 5 of our patients without LMNA mutation. The genetic defect associated with FPLD1 is currently unknown and some authors postulate that it is likely a more common FPLD than previously thought [19].

Another hypothesis for the 5 remaining women that underwent molecular biology studies and showed no mutations in the LMNA gene is the presence of PPARG mutations, found in FPLD3 phenotype. As described by Hegele et al., it appears that FPLD3, when compared with FPLD2, is associated with less extensive adipose loss, greater biochemical insulin resistance and more severe and earlier clinical end points, such as POS, hepatic steatosis, DM2 [20]. Our findings are consistent with this previous description, as depicted in Table 1.

FPLD2-affected women showed hypoleptinemia, insulin resistance and a more aggressive lipid profile when compared to control subjects. In general, there is a direct correlation between adipose mass and plasma leptin concentration [21]. The decreased plasma leptin in FPLD2 subjects results directly from the deficiency of peripheral adipose tissue mass. Consistent with another series, our results showed that the substantial, but incomplete, adipose tissue loss in FPLD2 is compatible with the observed reduction of mean plasma leptin to approximately 40% of normal [22].

The current study has several limitations. First, we were not able to search for another mutations linked to lipodystrophy phenotypes (PPARG, AKT2, and PLIN1). Therefore five out of 18 patients have not a genotype diagnosis. Second, our series only included female subjects and our findings are not generalizable to men. In addition, due to the rarity of the disease our small sample size limited our statistical analyses and correlations. Further studies of body composition comparing FLPD patients and controls are so warranted.

## Conclusion

In this study, the comparison of body composition with DXA in women with FPLD and healthy controls confirms a fat distribution pattern with marked reduction in lower extremities and gluteal region. A FMR cut-off point of 1.2 may be an objective and accurate index for the evaluation of FLPD2. These findings supports that the hallmark of FPLD2 is the lower-body fat reduction, that may be the most important characteristic associated with the increased cardio metabolic risk in these subjects.

## Abbreviations

FPLD: Familial Partial Lipodystrophy; FPLD2: Dunnigan-type Familial Partial Lipodystrophy; FLPD1: Kobberling-type Familial Partial Lipodystrophy; FPLD3: Familial Partial Lipodystrophy Type 3; DXA: Dual energy X-ray absorptiometry; POS: Polycystic Ovarian Syndrome; DM2: Type 2 Diabetes Mellitus; HDL-cholesterol: high-density-lipoprotein-cholesterol; HIV: Human immunodeficiency virus; LMNA: lamin A/C coding gene; PPARG: peroxisome proliferator-activated receptor coding gene; IFG: Impaired Fasting Glucose; CAD: Coronary Arterial Disease; HBP: High Blood Pressure; FMR: Fat Mass Ratio.

## Competing interests

The authors declare that they have no competing interests.

## Authors’ contributions

PBM carried out the immunoassay and participated in the sequence alignment; RSM carried out the molecular genetic studies, LZ participated in the design of the study and performed the statistical analysis, CMV, JEPO and AFG conceived of the study, and participated in its design and coordination and helped to draft the manuscript. All authors read and approved the final manuscript.
