# Erratum: Body composition study by dual-energy x-ray absorptiometry in familial partial lipodystrophy: finding new tools for an objective evaluation

**DOI:** 10.1186/s13098-015-0007-6

**Published:** 2015-03-14

**Authors:** Cynthia M Valerio, Lenita Zajdenverg, Jose Egidio P de Oliveira, Patricia B Mory, Regina S Moises, Amélio F Godoy-Matos

**Affiliations:** Metabolism Unit, Instituto Estadual de Diabetes e Endocrinologia, Rio de Janeiro and Catholic University, Rio de Janeiro, Brazil; Department of Nutrology, Universidade Federal do Rio de Janeiro, Rio de Janeiro, RJ Brazil; Division of Endocrinology, Universidade Federal de Sao Paulo, São Paulo, SP Brazil; Instituto Estadual de Diabetes e Endocrinologia, Rua Moncorvo Filho 90 – Centro, Rio de Janeiro, RJ CEP 20211-340 Brasil

Following publication of this manuscript [1] it was discovered that Dr Moises name had been inadvertently misspelled. This has now been corrected in the author list above. The authors apologise for any inconvenience this may cause.
